# Estimation of metabolic heat production and methane emission in Sahiwal and Karan Fries heifers under different feeding regimes

**DOI:** 10.14202/vetworld.2016.496-500

**Published:** 2016-05-24

**Authors:** Sunil Kumar, S. V. Singh, Priyanka Pandey, Narendra Kumar, O. K. Hooda

**Affiliations:** 1 Dairy Cattle Physiology Division, ICAR-National Dairy Research Institute, Karnal, Haryana, India; 2 Livestock Production and Management Division, ICAR-National Dairy Research Institute, Karnal, Haryana, India

**Keywords:** feeding regimes, heat production, Karan Fries, methane emission, Sahiwal

## Abstract

**Aim:**

The objective of this study was designed to estimate the metabolic heat production and methane emission in Sahiwal and Karan Fries (Holstein-Friesian X Tharparkar) heifers under two different feeding regimes, i.e., feeding regime-1 as per the National Research Council (NRC) (2001) and feeding regime-2 having 15% higher energy (supplementation of molasses) than NRC (2001).

**Materials and Methods:**

Six (*n* = 6) healthy heifers of Sahiwal and Karan Fries with 18-24 months of age were selected from Indian Council of Agricultural Research-National Dairy Research Institute, Karnal. An initial 15 days was maintained under feeding regime-1 and feeding regime-2 as adaptation period; actual experiment was conducted from 16^th^ day onward for next 15 days. At the end of feeding regimes (on day 15^th^ and 16^th^), expired air and volume were collected in Douglas bag for two consecutive days (morning [6:00 am] and evening [4:00 pm]). The fraction of methane and expired air volume were measured by methane analyzer and wet test meter, respectively. The oxygen consumption and carbon dioxide production were measured by iWorx LabScribe2.

**Results:**

The heat production (kcal/day) was significantly (p<0.05) higher in feeding regime-2 as compared to feeding regimen-1 in both breeds. The heat production per unit metabolic body weight was numerically higher in feeding regime-1 than feeding regime-2; however, the values were found statistically non-significant (p>0.05). The energy loss as methane (%) from total heat production was significantly (p<0.05) higher in feeding regime-1. The body weight (kg), metabolic body weight (W^0.75^), and basal metabolic rate (kcal/kg^0.75^) were significantly (p<0.05) higher in feeding regime-2 in both breeds.

**Conclusions:**

This study indicates that higher energy diet by supplementing molasses may reduce energy loss as methane and enhance the growth of Sahiwal and Karan Fries heifers.

## Introduction

The feeding system that meets animal’s energy requirements may result in the productivity of livestock to meet expectations of performance. The level of heat production and methane emission depends on type and level of feed intake. The heat production can be estimated from the oxygen (O_2_) consumed, carbon dioxide (CO_2_), and methane (CH_4_) produced by the animal using Brower’s equation. The level of metabolic heat production in domestic animals depends on their feed intake and muscular activity [1]. Increasing the energy levels and/or feeding additive concentrate supplements can improve energy efficiency and thus improve animal performance [2]. Methane is produced by the fermentation process, and it is considered as the inherent part of the energy metabolism in ruminants. Methane conversion rate (% of methane energy loss per gross energy intake) ranges from 8.4% to 10% in beef cattle [3]. The production rate of enteric methane can vary depending on the digestibility of the animals and the level of feed intake, breed, species, addition of lipid or ionophores to the feeds, alterations in microflora, and different animal activities [4]. Considerable efforts have been made to improve feed utilization and controlling ruminant methane emission [5]. Methane is formed during the fermentation of the feed in the rumen, and the amount is dependent on the quality and quantity of the diet.

The loss of ingested energy as eructated methane in cattle is around 6% [6], so it is necessary to find the feeding strategies that decrease methane emissions which would not only reduce the emissions of this greenhouse gases to the atmosphere but also improve the efficiency in terms of feed energy utilization in cattle production systems.

Since the interaction effects of energy intake levels on metabolic heat production and methane emission rate in Sahiwal and Karan Fries heifers has not been evaluated yet, so this study was carried out using Sahiwal and Karan Fries heifers to estimate the metabolic heat production and methane emission rate under different feeding regimes.

## Materials and Methods

### Ethical approval

The experiment was approved by the Institutional Animal Ethics Committee constituted as per the article No. 13 of the CPCSEA rules, laid down by Government of India.

### Study area

The experiment was conducted in the cattle yard of Indian Council of Agricultural Research-National Dairy Research Institute (ICAR-NDRI), Karnal, Haryana, India, altitude of 250 m above mean sea level and at 29°42′N latitude and 79°59′E longitudes. The highest temperature goes up to 45°C in summer and minimum temperature 3.5-4°C in winter. The average rainfall is about 700 mm.

### Animals and experimental design

Apparently 12 healthy Sahiwal and Karan Fries heifers (18-24 months) were selected from the Livestock Research Centre of ICAR-NDRI, Karnal, Haryana, India. The experimental animals were maintained as per the standard practices followed at the institute farm. The experimental animals were kept in separate shed throughout the experiment. Animals were let loose every week for exercise. The experiments on both groups of animals were conducted for 45 days under feeding regime-1 as per National Research Council (NRC), 2001 [7] and feeding regime-2 (15% higher energy level over and above the NRC, 2001 by supplementation of molasses) [7]. These animals were maintained on feeding regime-1 for 15 days continuously and later on shifted to feeding regime-2 (15% higher energy level) for next 15 days continuously. The experimental animals were given adaptation period of at least 15 days before actual experimentation. The feeds offered to the animals and residue left were recorded fortnightly interval to find out the total dry matter intake and *ad-libitum* water was given to the animals to find out the total water intake. All the animals before actual experimental work were trained to inspire and expire through a three-way valve and putting a face mask to collect expired gas and analyzed for O_2_ consumption, CO_2_, and methane (CH_4_) production. These animals were also trained to stand quietly in a wooden Travis for collection of gases before actual experiment.

### Collection of expired air sample

A three-way valve and face mask were used to collect expired air from experimental animals. The recording of O_2_ consumption and production of CO_2_ and CH_4_ was carried out for two days continuously in morning and evening after the end (15^th^ and 16^th^ day) of both the feeding regime.

The total expired air was collected in a Douglas bag at every half an hour interval for 4-5 min. The volume collected in Douglas bag was measured on wet test meter (precision scientific equipment U.S.A), and compositions (i.e., O_2_ and CO_2_) were analyzed on (iWorx LabScribe2), automatically the percentage of particular gas was displayed on LCD and memorized in the analyzer. CH_4_ was analyzed in expired air using methane analyzer (0.01-0.25%), Analytical Development Co., UK, ADC.

### Metabolic heat production

Metabolic heat production (kcal) was determined accurately from O_2_ consumption, CO_2_, and CH_4_ production. The following formula was used to determine metabolic heat production (H).

H = 3.866×O_2_ + 1.200×CO_2_ − 0.518×CH_4_ − 1.431×N [8].

Where,

H=Heat production (H, kcal)

O_2_=Oxygen consumption (L)

CO_2_=Carbon dioxide (L)

CH_4_=Methane production (L)

N = Quantity of urinary nitrogen excreted (g).

### Respiratory gas measurement

The rate of volume of expired air was calculated from formula:


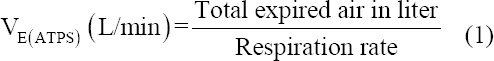


### Rate of O_2_ consumption

Rate of O_2_ consumption was calculated from formula:


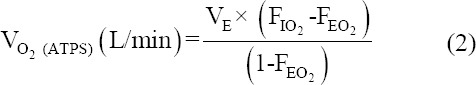


Where,

V_O2_=The rate of oxygen consumption

V_E_=The volume of air the subject breathes in 1 min (minute volume)

F_IO2_=The fraction (percentage divided by 100) of inspired air that is oxygen, i.e. 0.2094 (Since the percentage of oxygen in room air is constant at about 20.94%)

F_EO2_=The fraction of expired air that is O_2_ (i.e. the percentage measured with the oxygen analyzer).

### Rate of CO_2_ production

The Volume of CO_2_ produced per min was calculated using formula:


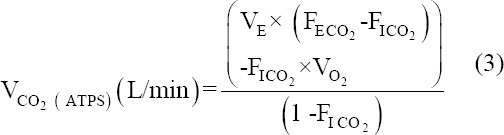


Where,

V_CO2_=The rate of CO_2_ production

V_E_ =The volume of air the subject breathes in 1 min (minute volume)

F_ECO2_=The fraction of expired air that is CO_2_

F_ICO2_=The fraction (percentage divided by 100) of inspired air that is CO_2_, i.e., F_ICO2_ = 0.0003 (Since a little percentage (0.03%) of CO_2_ in fresh air).

### Rate of CH_4_ production

The volume of CH_4_ produced per min was calculated:

V_CH4 (ATPS)_ (L/min)=V_E_×F_ECH4_ (4)

Where,

V_CH4_=The rate of methane production

V_E_= The volume of air the subject breathes in 1 min (minute volume)

F_ECH4_=The fraction of expired air that is CH_4_.

### Volume of standard temperature and pressure, dry air

The V_E_, V_O2_, V_CO2_, and V_CH4_ for standard temperature and pressure, dry air (STPD) obtained from respective V_E_, V_O2_, V_CO2_, and V_CH4_ for ambient temperature and pressure, saturated (ATPS) using following formula:

V_E_ (STPD) (L/min)=V_E_ (ATPS)×0.825 (5)

V_O2_ (STPD) (L/min)=V_E_ (STPD) (F_IO_−F_EO2_) (6)

V_CO2_ (STPD) (L/min)= V_E_ (STPD) (F_ECO2_−F_ICO2_) (7)

V_CH4_ (STPD) (L/min)=V_E_ (STPD)×(F_ECH4_) (8)

Where,

V_O2_=The rate of oxygen consumption

V_E_= The volume of air the subject breathes in 1 min (minute volume)

F_IO2_= The fraction (percentage divided by 100) of inspired air that is oxygen, i.e., 0.2094 (Since the percentage of oxygen in room air is constant at about 20.94%)

F_EO2_= The fraction of expired air that is oxygen (i.e., the percentage measured with the O_2_ analyzer)

V_CO2_=The rate of carbon dioxide production

F_ECO2_= The fraction of expired air that is carbon dioxide

F_ICO2_= The fraction (percentage divided by 100) of inspired air that is carbon dioxide, i.e. F_ICO2_ = 0.0003 (Since a little percentage (0.03%) of CO_2_ in fresh air)

V_CH4_=The rate of methane production

F_ECH4_=The fraction of expired air that is CH_4_

STPD= Standard temperature and pressure at dry air

ATPS= Ambient temperature and pressure at saturated air.

### Statistical analysis

The data analysis was carried out by SAS software, Version (9.1) of the SAS system [9]. The data were analyzed statistically for mean±standard error and analysis of variances.

## Results and Discussion

### Metabolic heat production

The result of O_2_ consumed, carbon dioxide (CO_2_), and methane (CH_4_) produced and metabolic heat production of Sahiwal and Karan Fries heifers during feeding regime-1 and -2 have been presented in Table-1. The mean body weight and metabolic body weight (W^0.75^) of Sahiwal heifers and Karan Fries heifers were significantly (p<0.01) higher in feeding regime-2 as compared to feeding regime-1. The O_2_ consumption, CO_2_ production, and metabolic heat production of Sahiwal and Karan Fries heifers were significantly (p<0.05) higher in feeding regime-2 as compared to feeding regime-1, whereas the metabolic heat production per unit metabolic body weight was higher in feeding regime-1.

**Table-1:** Metabolic heat production of Sahiwal and Karan Fries heifers during two different feeding regimes.

Parameters	Sahiwal		Karan Fries	
	
	Feed-1	Feed-2	Feed-1	Feed-2
Body weight (kg)	167.50±4.08	221.50±7.91**	221.83±15.63	297.00±13.47**
Metabolic body weight (kg)	46.54±0.85	57.38±1.53**	57.34±3.05	71.47±2.43**
Basal metabolic rate (kg)	3023.36±54.20	3710.45±96.89**	3707.61±193.51	4599.94±153.66**
Respiration rate (bpm)	21.50±0.56	22.83±0.40	24.33±0.98	26.33±0.88
Tidal volume (L)	2.27±0.07	2.18±0.07	2.20±0.13	2.17±0.12
Calculating time (min)	3.94±0.12	3.81±0.14	3.76±0.24	3.60±0.25
Volume in Douglas bag (L)	188.21±0.52	188.76±1.57	196.70±2.35	201.36±1.46
V_E_ (ATPS) (L/min)	48.83±1.50	49.91±2.02	53.39±3.36	57.17±3.66
V_O2_ (ATPS) (L/min)	1.00±0.03	1.17±0.04*	1.28±0.09	1.49±0.08
V_CO2_ (ATPS) (L/min)	0.89±0.01	0.99±0.03*	0.99±0.06	1.11±0.07
V_CH4_ (ATPS) (L/min)	0.05±0.001	0.04±0.005	0.08±0.008	0.07±0.009
VE (STPD) (L/min)	40.29±1.24	41.17±1.67	44.04±2.77	47.17±3.01
V_O2_ (STPD) (L/min)	0.82±0.02	0.96±0.03*	1.06±0.07	1.23±0.07
V_CO2_ (STPD) (L/min)	0.73±0.008	0.81±0.030*	0.82±0.05	0.92±0.06
V_CH4_ (STPD) (L/min)	0.04±0.001	0.03±0.004	0.06±0.007	0.06±0.007
HP/min (kcal)	4.06±0.11	4.70±0.17*	5.31±0.21	5.99±0.21*
HP/day (kcal)	5858.01±163.30	6779.90±250.37*	7653.67±304.36	8634.67±312.91*
HP/metabolic body weight (kcal/kg^0.75^)	125.92±3.23	118.20±3.48	134.83±7.20	120.76±0.43

* p<0.05 and

** p<0.01 differ significantly at 5% and 1% level, respectively, within breed.

STPD=Standard temperature and pressure, ATPS=Ambient temperature and pressure, HP=Heat production

In this metabolic heat production study, heat production partition was used as an index to determine heat (energy) retention. The result of this study demonstrated that cattle fed a diet that contains higher energy level (15% by the supplementation of the molasses) according to their metabolic body size showed improved energy efficiency and energy retention and thus improved the growth rate [10]. A consequence of this greater energy retention was a decreased proportion of energy intake to energy losses in methane emission and heat production when the energy level was increased. These results indicated that increasing energy level is an important factor in improved body weight gain. The O_2_ consumption, CO_2_, and CH_4_ production during both the feeding regimes are the determinant factors for the metabolic heat production. This difference in O_2_ consumption, CO_2_, and CH_4_ production was mainly due to higher energy content during feeding regime-2. Tiwari *et al*. [11] reported that the overall daily means per unit body metabolic body weight (kgw^0.75^) for O_2_ consumption, CO_2_ and CH_4_ production, and heat production were 17.03 L, 11.7 L, 0.12 L, and 331 KJ, respectively, in growing buffalo calves, which indicated that a low-quality diet increased energy loss. Sauvant and Giger-Reverdin [12] suggested that feeding level influenced the ration of ME to GE, indicating that increased energy level or molasses supplementation could improve the energetic efficiency of animals. Further, recent research in a temperate zone [2] indicated that increasing the energy level and/or feeding additive concentrated supplements can improve energetics efficiency and thus improves animal growth and performance.

This study indicated that an increased in energy intake increased the energy efficiency and reduced the metabolic heat production per unit metabolic body weight. It also indicated that Sahiwal produces less metabolic heat production as compared to Karan Fries heifers that confer the ability of the indigenous breed to withstand the hot, humid condition of the tropical zone is higher than the crossbred animals.

### Energy loss as methane

The results of methane (CH_4_) emission, CH_4_ emission rate in Sahiwal and Karan Fries heifers under feeding regime-1 and -2 have been presented in Table-2. The level of energy loss as methane in Sahiwal and Karan Fries heifers was found to be higher during feeding regime-1 as compared to feeding regime-2. Methane emission rate, the proportion of gross energy intake that is released as enteric methane energy (% GEI), is a critical factor used to assess the potential extent of global warming in national inventories and enteric methane estimation [13]. These experimental results suggest that the methane emission rate (ranging from 7.3% to 11.5%) of Brahman cattle that are maintained in a tropical beef production feeding system [2]. Johnson and Johnson [6] also reported a methane energy loss of 6-7% of gross energy intake when forage was fed, and this reduces to 2-3% when high grain concentrate was offered at near *ad-libitum* intake level.

**Table-2:** Methane emission by Sahiwal and Karan Fries heifers during two different feeding regimes.

Parameters	Sahiwal		Karan Fries	
	
	Feed-1	Feed-2	Feed-1	Feed-2
Body weight (kg)	167.50±4.08	221.50±7.91**	221.83±15.63	297.00±13.47**
Metabolic body weight (kg)	46.54±0.85	57.38±1.53**	57.34±3.05	71.47±2.43**
Basal metabolic rate (kg)	3023.36±54.20	3710.45±96.89**	3707.61±193.51	4599.94±153.66**
Mean% CH_4_	0.10±0.001	0.09±0.009	0.15±0.012	0.13±0.008
V_CH4_ (ATPS) (L/min)	0.05±0.001	0.04±0.005	0.08±0.008	0.07±0.009
V_CH4_ (STPD) (L/min)	0.04±0.001	0.03±0.004	0.06±0.007	0.06±0.007
CH_4_ (L/day)	62.20±1.74	56.93±7.01	100.55±10.59	92.49±11.32
Energy loss as CH_4_ (kcal/min)	0.40±0.01	0.37±0.04	0.65±0.06	0.60±0.07
Energy loss as CH_4_ (kcal/day)	587.81±16.46	538.03±66.32	950.26±100.12	874.06±107.00
CH_4_*9.45 (kcal)/HP (kcal/min)	10.04%±0.18	7.85%±0.81*	12.36%±1.02	9.98%±0.91

* p<0.05 and

** p<0.01 differ significantly at 5% and 1% level, respectively, within breed.

STPD=Standard temperature and pressure, ATPS=Ambient temperature and pressure, HP=Heat production

The result of this study was in consistent of Hammond *et al*. [14] that reported a high methane emission rate of cattle fed high fiber feedstuff based diets. The typically poor quality feed available in our tropical condition may be the main factor affecting methane emission rate. Improving feed quality by supplementation with feed additive, i.e., molasses or increasing the energy level of the ration of the animals was helpful in reduction in methane emission rate. Our results indicate that digestibility of feedstuffs and rate of passage may increase with increasing energy level of the feed that decrease the opportunity for degradation of potentially degradable NDF; consequently, methane emission rate is decreased [15]. The result of this study are in accordance to those of Chaokaur *et al*. [10], who reported 8% and 11.5% energy loss as methane when animals were maintained on higher and lower level of feeding, respectively. This study also indicated that the methane emission rate was more in Karan Fries than Sahiwal heifers. This indicates that our indigenous breeds are more adapted to our climatic condition than the crossbred and impart less global warming.

## Conclusion

Based on this study, it can be concluded that feeding of extra energy (15%) by the addition of molasses in the feed of Sahiwal and Karan Fries heifers helped in reduction of metabolic heat production/W^0.75^ and energy loss in terms of methane production. This reduction in enteric methane emission loss will be helpful in reduction of global warming.

The study further showed that Sahiwal heifers contribute less methane emission to the environment as compared to Karan Fries (crossbred) maintained on the same feeding schedule, indicating the importance of zebu cattle toward the lesser global warming. By improving the feeding of ruminant animals may help in two ways, i.e., more availability of energy for productive process and lesser global warming.

## Authors’ Contributions

SVS has planned the study. SK conducted the experiment and recorded the O_2_, CO_2_, and CH_4_ emission in expired air. SK and PP estimated the metabolic heat production and methane emission based on recorded data. SK, SVS, and OKH performed statistical analysis. SK and NK drafted and revised the manuscript under the guidance of SVS. All authors read and approved the final manuscript.
